# Impact of transforming karst mountainous forests into urban parks on plant diversity patterns

**DOI:** 10.1002/ece3.70194

**Published:** 2024-08-15

**Authors:** Weize Wang, Xiaoyan Gao, Chunhua Cen, Mengping Jian, Zijin Wang, Jingyi Yang

**Affiliations:** ^1^ College of Forestry Guizhou University Guiyang China

**Keywords:** homogenization, native species, regeneration, transformation, urban mountainous forests, woody plants

## Abstract

The conversion of urban montane forest resources into urban parks requires careful assessment to understand its impacts on plant diversity over time. This study aims to enhance urban biodiversity conservation strategies by analyzing how habitat type and park age affect woody plant diversity. We surveyed woody plant species in 60 sample plots across two different habitats (remnant forest vs. artificial green space) within three mountain parks in Guiyang, China, established at different times. The alpha diversity of saplings/seedlings was significantly higher in remnant forests than in artificial green spaces. Artificial green spaces exhibited more homogenous woody plant composition compared with remnant forests. Newer parks showed greater variation in plant composition between the two habitats than older parks. Indicative species in remnant forests were predominantly native, whereas those in artificial green spaces were mainly ornamental species. The transformation of karst mountainous forests into urban parks leads to the homogenization of woody plant composition and impedes the regeneration of saplings/seedlings. Therefore, it is crucial to manage these conversions carefully and strive to preserve as many native species as possible to support urban plant diversity conservation.

## Graphical abstract

In this article, we investigated the impact of converting karst mountainous forests into urban parks on woody plant diversity and composition in a subtropical Chinese city undergoing rapid urbanization. Our research distinguishes itself from previous work in two main ways: first, we assessed the differences in diversity and species composition of woody plants across three distinct parks and between two types of habitats. Second, we explored variations in indicator species across these habitats—a focus not previously reported.

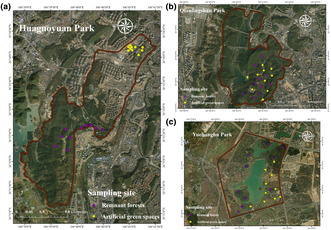

## 1 INTRODUCTION

Karst terrain, shaped by the interaction of rainfall and groundwater with carbonate bedrock such as limestone and dolomite, comprises a unique landscape noted for its ecological significance (Zhang et al., 2010). These landscapes are vital hotspots for biodiversity conservation, characterized by specialized habitats, diverse vegetation, and rich biodiversity (Du et al., 2017; Zhang et al., 2010). Southwest China, recognized as one of the world's largest karst regions, is notably the most extensive and concentrated ecologically fragile area globally (Brandt et al., 2018; Du et al., 2019; Hu et al., 2023; Wu et al., 2022). Situated in the heart of Guizhou Province, Guiyang serves as a prime example of a karst mountain city. It is endowed with a robust base of residual mountain forests and a well‐protected ecological environment (Liao et al., 2022; Yang et al., 2021). The rapid urbanization in Guiyang, however, poses significant threats to these forest resources through habitat loss and fragmentation, alongside the decline in native species (Huang et al., 2013; Ramalho et al., 2014). In response to these challenges, Guiyang has established numerous parks aimed at mitigating urban encroachment on forested areas, preserving biodiversity within urban ecosystems, and enriching recreational opportunities for residents (Luo et al., 2022). Despite these initiatives, the impacts of parkland renovation on both remnant forests and artificial green spaces have yet to be sufficiently examined.

The distinction between remnant forests and artificial green spaces is significant. Remnant forests are natural or semi‐natural woodlands in urban settings that have been preserved throughout urban development (Zipperer, 2002). These forests act as crucial reservoirs for native species and as biodiversity hotspots within urban ecosystems (Foo, 2016; Kowarik & von der Lippe, 2018). Subject to less human interference than their artificial counterparts, remnant forests maintain their natural or near‐natural habitats, safeguarding the survival of native species and providing diverse, irreplaceable ecosystem services to urban areas (Chen et al., 2021). Although the abundance of native species in these forests is lower than that of introduced species, they are essential for supporting urban biodiversity (Freitas et al., 2020). In contrast, artificial green spaces are constructed through deliberate human intervention and modification. Typically found in parks, these spaces predominantly feature plant species selected for their ornamental value, enhancing the landscape's esthetic appeal. Such environments are primarily designed for recreation and leisure, intentionally crafted to garner public appreciation (Barrico et al., 2018). Due to the high levels of human disturbance and significant alterations from their natural settings, these spaces often become havens for exotic species (Gong et al., 2013). The introduction of ornamental plants not only increases the botanical variety but also plays a vital role in the sustainability of urban ecosystems (Ren et al., 2017).

The duration of development of urban mountain parks profoundly influences the plant diversity within both remnant forests and artificial green spaces, as well as the distinctions between these environments. Over time, the species richness in remnant forests within parks tends to naturally increase, whereas artificial green spaces are populated with plant species that reflect the landscaping preferences of different periods (Figueroa et al., 2018). Consequently, it can be generally inferred that the longer a park has been in development, the greater and more stable its plant species richness becomes. During the initial phases of park construction, introducing new plant species can enhance floral diversity (Lososová et al., 2012; Ren et al., 2017). However, as parks age, there tends to be a decline in native plant populations, while the numbers of exotic species rise significantly (Fischer et al., 2016; Nielsen et al., 2014). This trend towards the dominance of introduced species in artificial settings can lead to biological homogenization (Gong et al., 2013). Conversely, the application of advanced management techniques over time can lead to a more diverse species composition, reflecting the evolution of park conservation strategies (Fischer et al., 2016). Despite these observations, there remains a scarcity of research explicitly addressing the impact of park development duration on plant diversity.

In recent years, the accelerated pace of urbanization has increasingly underscored the importance of urban parks. These parks not only enhance outdoor recreation and leisure opportunities for city residents but also confer notable health benefits, both physical and mental (Chen et al., 2023; Ward Thompson et al., 2016). Prior research has primarily focused on the landscape design of urban parks and the assessment of their plant diversity (Ma et al., 2023; Tomitaka et al., 2021). Additionally, urban parks play a crucial role in conserving plant diversity within cities by providing habitat and creating microclimates that facilitate plant growth (Elgizawy, 2014). Research has shown that plant diversity in urban parks is influenced by several factors, including the surrounding landscape pattern, urbanization level, and park size (Chang et al., 2021; Chen et al., 2023; Ma et al., 2023). Findings indicate that larger parks support higher plant diversity and are more conducive to the conservation of native species (Figueroa et al., 2018). Furthermore, parks situated in highly urbanized areas have been shown to exhibit increased plant diversity (Clarke et al., 2013). However, urban mountain parks have received less attention, and the impact of converting remnant mountains into parkland on plant diversity remains poorly understood. Additionally, although extensive studies exist on parks in flat urban areas and major cities such as Beijing, Shanghai, and Hangzhou, research on parks in karst mountain cities remains sparse (Hu et al., 2022; Qin et al., 2021; Shen et al., 2017).

In this study, we analyzed three mountain parks constructed in different years within Guiyang, China. Each park was segmented into two distinct habitats: remnant forest and artificial green space. The primary aim of this research was to assess the impact of park age and habitat type on plant diversity. This study addresses the unique challenge in karst cities, where extensive remnant forests, shaped by distinct terrain and climate, are transformed into parks to meet recreational needs. It specifically investigates the underexplored impact of converting remnant mountains into parkland on plant diversity. By focusing on Guiyang's urban parks and analyzing plant diversity across remnant forests and artificial green spaces, this research provides novel insights into the ecological implications of park development in karst mountain environments. The specific objectives were (1) to evaluate differences in diversity and species composition of woody plants across the three parks and between the two habitat types and (2) to investigate the variation in indicator species between the two habitats. We hypothesized that converting karst mountainous forests into urban parks can lead to the homogenization of woody plant composition due to the introduction of ornamental species and the alteration of the natural habitat. This process favors species that thrive under uniform, managed conditions typical of artificial green spaces, thereby reducing native plant diversity.

## 2 MATERIALS AND METHODS

### 2.1 Study area

**FIGURE 1:**
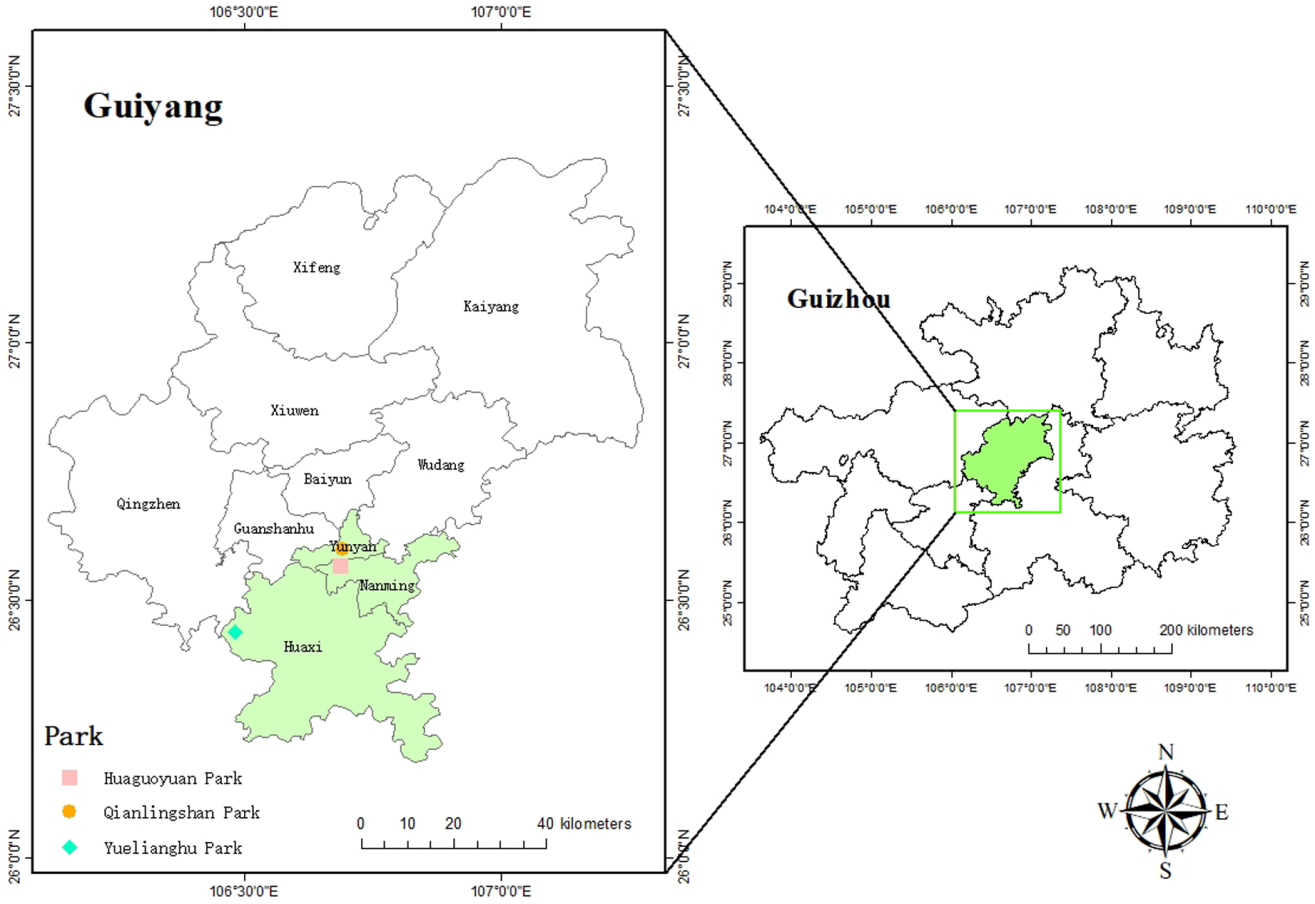
The study area, including the three park locations.

The study examines Guiyang City, a principal urban center in southwest China and the capital of Guizhou Province (Figure 1). Located on the Yunnan‐Guizhou Plateau, Guiyang features a landscape predominantly characterized by karst topography, including numerous mountains and hills, distinguishing it as an exemplary karst mountain city in China. The region's climate is classified as subtropical monsoon, with the primary vegetation being subtropical evergreen deciduous broad‐leaved mixed forests. In natural forest stands, the dominant canopy species primarily belong to the Fagaceae and Lauraceae families, with the Theaceae and Magnoliaceae families also being prevalent. The study area encompasses two main soil types: limestone soil and yellow soil. Since 1996, Guiyang has undergone rapid urban expansion. The city's steep topography has facilitated the preservation of substantial mountain forest resources within its limits during this urbanization process (Yang et al., 2021). These remnant forests have been strategically converted into urban mountain parks to meet the recreational and leisure needs of the urban population while conserving urban biodiversity. Guiyang was selected as the focal point for this research due to its diverse range of parks, which vary in type and developmental era, and its location within a recognized biodiversity hotspot (Liu et al., 2018; Long & Shi, 2021).

### 2.2 Field survey

**FIGURE 2:**
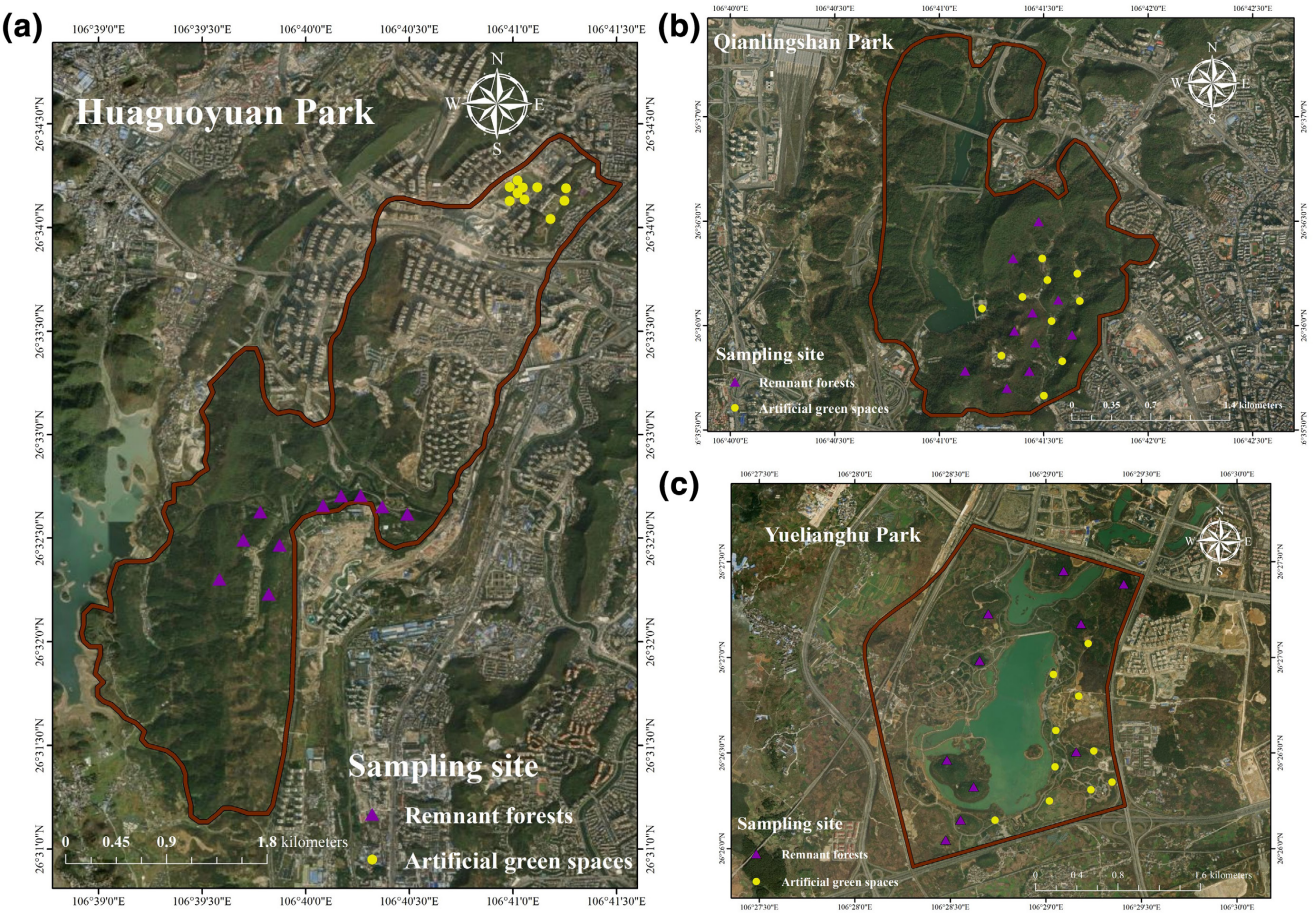
Spatial location of sample plots in (a) Huaguoyuan, (b) Qianlingshan and (c) Yuelianghua park.

In this study, urban mountain parks are defined as parks situated within urban boundaries that incorporate mountains or mountainous terrain features. A combination of online research and field visits facilitated the selection of three such parks for analysis: Qianlingshan Park (established in 1957), Huaguoyuan Park (established in 2010), and Yuelianghu Park (established in 2020), as shown in Figure 1. Each park represents a case of transformed and expanded urban space through the integration of remnant mountain forests, a significant portion of which has been preserved within these parks. The research further categorized the landscapes within these parks into two distinct habitats: artificial green spaces and remnant forests. To conduct a detailed analysis, 10 sample plots measuring 20 × 20 meters were randomly established in each habitat type within the parks, resulting in 20 sample plots per park and a total of 60 sample plots across the study (Figure 2). Table 1 presents some basic information about the three parks.

**TABLE 1:** Basic information of three parks.

Park	Established time	Remaining forest area (km^2^)	Artificial green space area (km^2^)	Major plant species
Qianlingshan park	1957	4.0546	0.2235	*Itea yunnanensis* *Lindera communis* *Camellia oleifera* *Myrsine africana* *Osmanthus fragrans* *Cerasus yedoensis* *Ginkgo biloba* *Rhododendron simsii* *Fatsia japonica*
Huaguoyuan park	2010	5.473	0.1826	*Itea yunnanensis* *Lindera communis* *Alangium chinense* *Rosa cymose* *Osmanthus fragrans* *Cerasus yedoensis* *Rhododendron simsii* *Ligustrum japonicum* *Euonymus japonicus*
Yuelianghu park	2020	0.9499	2.9021	*Itea yunnanensis* *Populus adenopoda* *Corylus heterophylla* *Myrsine africana* *Osmanthus fragrans* *Cerasus yedoensis* *Rhododendron simsii* *Photinia × fraseri*

### 2.3 Data collection

Between June and August 2023, a survey of woody plants was conducted across 60 sample plots. During this period, the species and abundance of all woody plants within each plot were meticulously recorded. These plants were classified according to the Flora of Guizhou (Chen, 2004) to discern whether they were trees or shrubs. Further, trees were differentiated into two categories: adult trees, defined as those with a diameter at breast height (DBH) greater than 3 cm, and saplings/seedlings. This classification facilitated an exploration of the different impacts on the growth stages of the trees, as noted in recent research (Yang et al., 2022). Subsequently, the data collected from the three parks were aggregated and organized into six distinct categories, reflecting both the park and habitat type: Qianlingshan‐Remnant (Q1), Qianlingshan‐Artificial (Q2), Huaguoyuan‐Remnant (H1), Huaguoyuan‐Artificial (H2), Yuelianghu‐Remnant (Y1), and Yuelianghu‐Artificial (Y2). This methodical categorization allowed for a nuanced analysis of species distribution and vegetation dynamics across different urban park settings.

### 2.4 Data analysis

This study was conducted to explore variations in the alpha diversity of woody plants across three parks and two habitat types, using a range of diversity indices. Alpha diversity, a concept reflecting species richness and evenness within a habitat (Thukral, 2017), was quantified using the species richness index, Simpson index, Shannon‐Wiener index (hereafter, Shannon index), and Pielou index. Differences in alpha diversity among six groups were assessed using the Wilcoxon signed‐rank test, a nonparametric statistical method. The results were visually presented through error bar graphs. Additionally, to examine potential discrepancies in species composition across the parks and habitat types, an Analysis of Similarities (ANOSIM) was conducted. This technique, a nonparametric multivariate analysis, is analogous to a one‐way ANOVA. Subsequent analysis involved Principal Coordinate Analysis (PCoA) to elucidate the species composition differences among six distinct categories. We also calculated indicator values, ranging from 0 to 1, based on species abundance in both remnant forests and artificial green spaces. Solely species with significant indicator values (*p*‐value < .05) were deemed indicative of a particular habitat type. All data analyses were performed using the R software, version 4.2.3 (R Core Team, 2019).

## 3 RESULTS

### 3.1 The alpha diversity of woody plants in three parks

**FIGURE 3:**
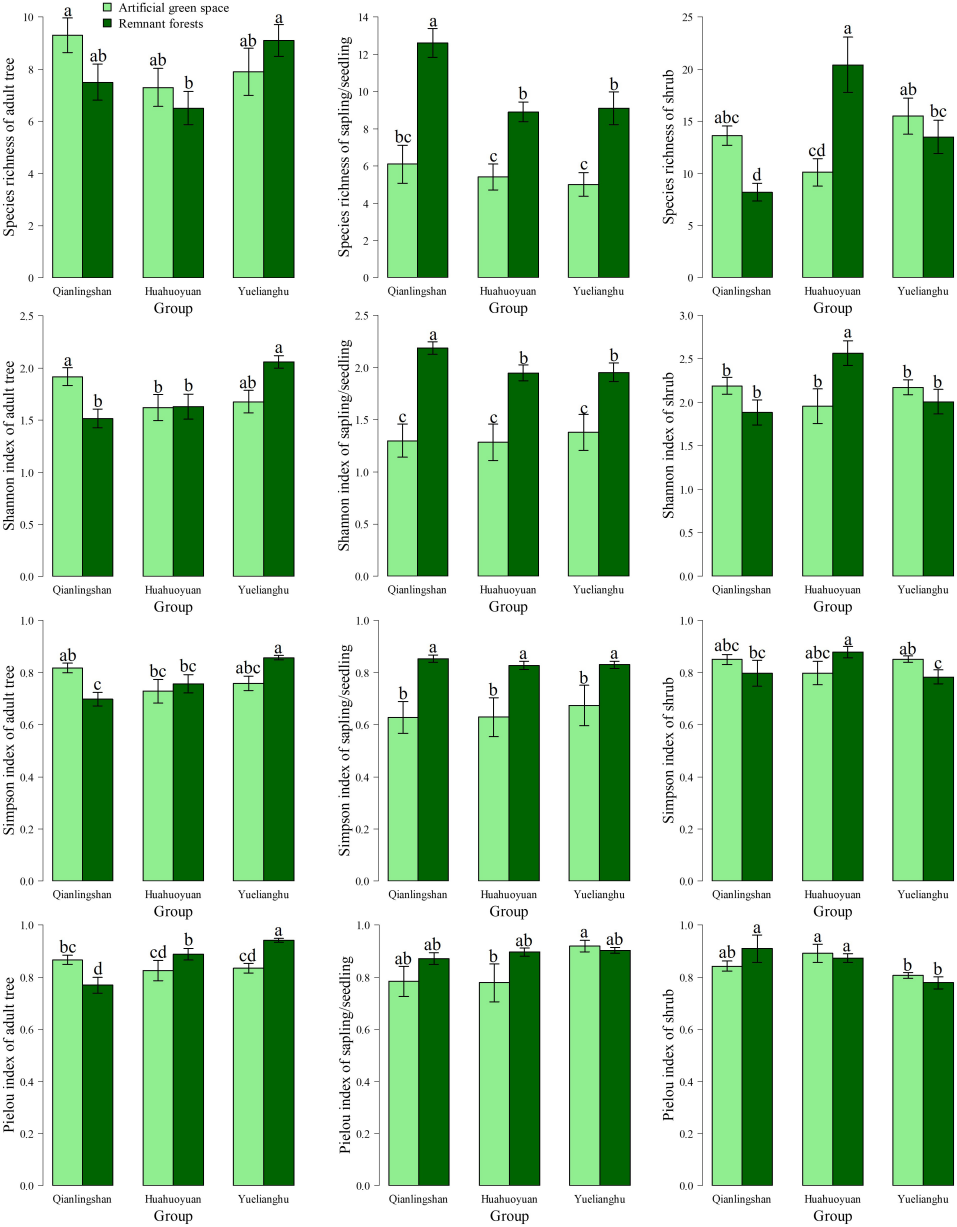
Alpha diversity of woody plant species. Different characters are used to represent significant differences (*p* < .05), whereas the same characters indicate non‐significant differences (*p* > .05).

A survey of the woody plants was conducted across 60 sample plots within three parks. In total, 128 tree species and 121 shrub species were identified. These included 104 species of adult trees and 100 species of saplings/seedlings. The Wilcoxon test results (Figure 3) demonstrate that remnant forests have the highest sapling/seedling species richness across all three parks, exceeding that in artificial green spaces. In the adult tree layer, Yuelianghu Park's remnant forest shows the greatest species richness, with no significant differences among artificial green spaces. Qianlingshan Park's remnant forest displays significantly higher species richness in saplings/seedlings than the other parks, without notable differences in their artificial green spaces. In the shrub layer, Huaguoyuan Park's remnant forest surpasses the others in species richness.

### 3.2 Compositional dissimilarity across three parks with two habitat types

The ANOSIM results (Table 2) show highly significant differences in species composition between habitat types and among the three parks (*p* < .01). Artificial green spaces had more homogenous woody plant compositions compared with remnant forests. Yuelianghu Park exhibited the most distinct disparity in species composition between its remnant forests and artificial green spaces.

**TABLE 2:** Results of the analysis of similarity among different groups.

Vegetation type	Comparative groups	ANOSIM statistic *R*	Significance
Adult tree	Q1, Q2	.586	.001
	H1, H2	.477	.001
	Y1, Y2	.801	.001
	Q1, H1, Y1	.538	.001
	Q2, H2, Y2	.217	.001
Sapling/seedling	Q1, Q2	.355	.001
	H1, H2	.376	.001
	Y1, Y2	.762	.001
	Q1, H1, Y1	.648	.001
	Q2, H2, Y2	.329	.001
Shrub	Q1, Q2	.881	.001
	H1, H2	.411	.001
	Y1, Y2	.992	.001
	Q1, H1, Y1	.602	.001
	Q2, H2, Y2	.466	.001

*Note*: ANOSIM statistic R approaching 1 indicates greater dissimilarity between groups, thus highlighting increased distinctions among the groups. Q1: remnant forests in Qianlingshan; Q2: artificial green spaces in Qianlingshan; H1: remnant forests in Huaguoyuan; H2: artificial green spaces in Huaguoyuan; Y1: remnant forests in Yuelianghu; Y2: artificial green spaces in Yuelianghu.

**FIGURE 4:**
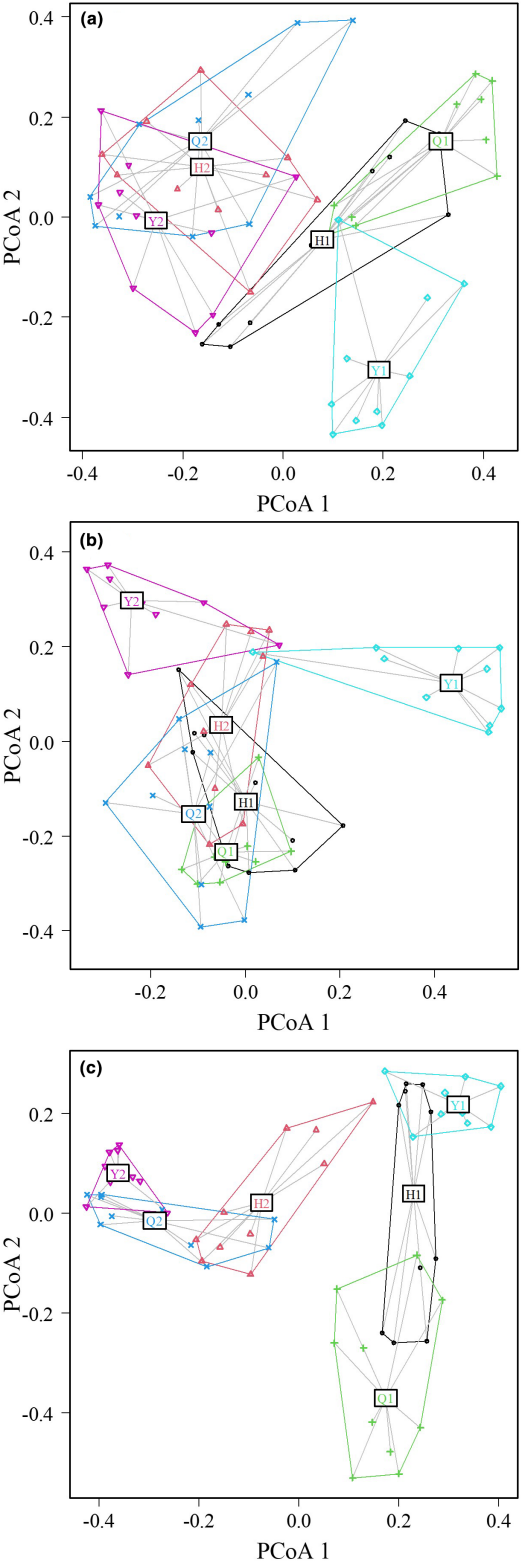
Principal coordinate analysis plots were generated to compare the species composition in the adult tree layer (a), sapling/seedling layer (b), and shrub layer (c) across six different groups. Increased overlap between groups suggests a higher level of similarity in species composition. Q1: remnant forests in Qianlingshan; Q2: artificial green spaces in Qianlingshan; H1: remnant forests in Huaguoyuan; H2: artificial green spaces in Huaguoyuan; Y1: remnant forests in Yuelianghu; Y2: artificial green spaces in Yuelianghu.

As shown in Figure 4, in the adult tree and shrub layers (Figure 4a,c), artificial green spaces and remnant forests in the three parks show no overlap, indicating significant dissimilarities. Artificial green spaces across the parks exhibit a higher similarity with each other than with remnant forests. In the sapling/seedling layer (Figure 4b), there is some species composition overlap between the artificial green spaces and remnant forests in Qianlingshan Park and Huaguoyuan Park, with Yuelianghu Park showing less overlap.

### 3.3 Indicator species for two habitat types

Table 3 summarizes the indicator species identified in the remnant forests and artificial green spaces of the three parks. In the remnant forests, *Itea yunnanensis* emerged as the most significant indicator species in both the adult tree and the sapling/seedling layers. Additionally, in the shrub layer, the most notable indicator species included *Myrsine africana*, *Smilax china*, and *Zanthoxylum armatum*. Conversely, in the artificial green spaces, *Osmanthus fragrans* was the predominant indicator species in both the adult and seedling/sapling layers. The most significant indicator species in the shrub layer were *Loropetalum chinense var. rubrum*, *Fatsia japonica*, *Photinia × fraseri*, and *Ligustrum sinense*.

**TABLE 3:** Indicator species in remnant forests and artificial green spaces.

Remnant forest			Artificial green space		
Indicator species	Stat	*p*‐value	Indicator species	Stat	*p*‐value
*Itea yunnanensis* (1)	.683	.001***	*Osmanthus fragrans* (1)	.796	.001***
*Lindera communis* (1)	.548	.003**	*Cerasus yedoensis* (1)	.606	.002**
*Corylus heterophylla* (1)	.447	.015*	*Prunus cerasifera* (1)	.544	.002**
*Populus adenopoda* (1)	.447	.025*	*Ginkgo biloba* (1)	.544	.003**
*Diospyros kaki* (1)	.428	.050*	*Acer palmatum* (1)	.516	.007**
*Rhamnella franguloides* (1)	.408	.048*	*Lagerstroemia indica* (1)	.505	.046*
*Itea yunnanensis* (2)	.722	.001***	*Punica granatum* (1)	.447	.026*
*Alangium chinense* (2)	.548	.001**	*Osmanthus fragrans* (2)	.681	.001***
*Citrus reticulata* (2)	.508	.034*	*Cerasus yedoensis* (2)	.447	.027*
*Camellia oleifera* (2)	.483	.016*	*Morus alba* (2)	.447	.024*
*Lindera communis* (2)	.483	.009**	*Sapium sebiferum* (2)	.447	.020*
*Rhus chinensis* (2)	.483	.006**	*Loropetalum chinense var. Rubrum* (3)	.842	.001***
*Populus adenopoda* (2)	.447	.022*	*Fatsia japonica* (3)	.785	.001***
*Corylus heterophylla* (2)	.447	.025*	*Photinia × fraseri* (3)	.773	.001***
*Rhamnella franguloides* (2)	.447	.018*	*Ligustrum sinense* (3)	.733	.001***
*Koelreuteria paniculate* (2)	.445	.043*	*Aucuba japonica var. variegata* (3)	.683	.001***
*Myrsine africana* (3)	.825	.001***	*Rhododendron pulchrum* (3)	.652	.001***
*Smilax china* (3)	.760	.001***	*Pittosporum tobira* (3)	.562	.030*
*Zanthoxylum armatum* (3)	.717	.007**	*Boehmeria nivea* (3)	.548	.002**
*Rosa cymose* (3)	.687	.001***	*Rubus idaeus* (3)	.508	.041*
*Rubus coreanus* (3)	.615	.024*	*Ligustrum japonicum* (3)	.483	.014*
*Zanthoxylum scandens* (3)	.606	.001***	*Euonymus japonicus* (3)	.483	.010**
*Lonicera japonica* (3)	.602	.004**	*Rhododendron simsii* (3)	.447	.019*
*Hypericum monogynum* (3)	.577	.002**	*Jasminum nudiflorum* (3)	.408	.039*
*Sageretia thea* (3)	.577	.025*			
*Pyracantha fortuneana* (3)	.554	.023*			
*Serissa japonica* (3)	.548	.003**			
*Lespedeza bicolor* (3)	.542	.002**			
*Toddalia asiatica* (3)	.534	.009**			
*Acanthopanax trifoliatus* (3)	.524	.020*			
*Coriaria nepalensis* (3)	.483	.042*			
*Rhamnus leptophylla* (3)	.483	.010**			
*Rhamnus heterophylla* (3)	.447	.023*			
*Rubus parvifolius* (3)	.408	.045*			
*Lespedeza mucronate* (3)	.408	.045*			
*Zanthoxylum nitidum* (3)	.408	.045*			

*Note*: The numbers inside the parentheses represent different types of plants: 1 for adult tree, 2 for sapling/seedling, and 3 for shrub.

* *p* < .05

** *p* < .01

*** *p* < .001.

## 4 DISCUSSION

Previous research on urban parks has mainly focused on flat areas and artificial green spaces, with limited attention to how park development time affects plant diversity. This study investigates urban mountain parks in karst regions, comparing woody plant diversity between artificial green spaces and residual forests, and assessing the impact of park transformation over time. The findings indicate significant differences in alpha diversity and species composition between the two habitats. Parks with shorter development times showed lower species similarity, while similarity gradually increased with longer park development durations.

The study indicated that plant diversity among saplings/seedlings in remnant forests exceeded that in artificial green spaces across the three parks analyzed. Saplings/seedlings, more susceptible to external disturbances compared with adult trees, face numerous natural and anthropogenic threats that impede their growth and survival (Nilsson & Wardle, 2005; Ribeiro et al., 2016). Artificial green spaces, designed primarily for urban recreation, experience a higher frequency of human activity compared with remnant forests, adversely affecting the saplings/seedlings found therein. Moreover, the intentional configuration of urban parks to maximize recreational areas for city residents often results in the constriction of these spaces into smaller patches. This limitation on space and soil resources further inhibits the establishment and thriving of saplings/seedlings (Chang et al., 2021; Rusterholz et al., 2020).

Contrary to expectations, the species richness of woody plants within the three parks did not increase over time, likely due to anthropogenic disturbances. The moderate disturbance hypothesis (Connell, 1978) suggests that species richness and diversity peak at moderate disturbance levels. Species richness may initially rise after park construction but then decrease over time (Liu et al., 2019; Wu et al., 2024). These findings imply that urban park development impacts woody plants less significantly than anthropogenic disturbances. Among the parks, Yuelianghu Park, the most recently constructed, had the lowest species composition similarity between habitat types, whereas Huaguoyuan Park had the highest. Initially, the introduction of cultivated garden plants increases dissimilarity between parks' artificial green spaces and remnant forests (Gong et al., 2013; Ren et al., 2017). However, species exchange over time tends to increase their similarity.

The artificial green spaces in all three parks exhibited a higher degree of similarity compared with the remnant forests. This increased similarity in artificial green spaces is due to the deliberate planting of uniform ornamental garden species. In contrast, remnant forests have more complex structures and stable habitats, supporting a wider diversity of biological communities (Fahey & Casali, 2017). Additionally, remnant forests enhance species diversity through favorable microclimate conditions (Koelemeijer et al., 2021). These factors contribute to the lower similarity among remnant forests. In natural settings, similarity is consistent across the three primary vegetation types. However, in artificially planted green spaces, similarity is highest in the adult tree layer and lowest in the shrub layer. This pattern arises from anthropogenic selection, where vegetation is chosen based on esthetic, functional, socio‐cultural, and economic considerations. As a result, a limited range of plant species fulfilling these roles are predominantly planted (Acar et al., 2007; Hagwet et al., 2024; Kendal et al., 2014). In contrast, shrubs generally exhibit greater diversity and adaptability.

A comparative analysis of species composition across six categories, incorporating three parks and two habitat types, revealed that the dissimilarities in the species composition of the adult tree and shrub layer between remnant forests and artificial green spaces were more pronounced compared with those in the sapling/seedling layer. In urban environments, saplings/seedlings are particularly vulnerable to both anthropogenic and natural factors (Lv et al., 2023). Concurrently, anthropogenic disturbances facilitate the proliferation and establishment of exotic garden plants in artificial green spaces (Hobbs & Huenneke, 1992). With the progression of park development, the potential for woody plants in artificial green spaces and remnant forests to spread and disperse via wind, animal transport, and human activities increases (Palliwoda et al., 2017). As a result, the species composition of saplings and seedlings in Qianlingshan Park and Huaguoyuan Park showed a high degree of overlap, whereas Yuelianghu Park, having a shorter developmental period, displayed a lower degree of overlap in this layer compared with the other parks.

The indicator species analysis revealed a clear distinction between species observed in remnant forests and those in artificial green spaces. Specifically, the indicator species in the remnant forests were native, while those in the artificial green spaces consisted entirely of garden species prized for their ornamental value. This observation aligns with our expectations. Common species in the three parks, such as *O. fragrans*, *C. yedoensis*, *L. chinense var. rubrum*, and *F. japonica*, have been extensively utilized as ornamental landscape plants. The prevalence of these ornamental species in urban parks significantly contributes to the high proportion of nonnative species (Nielsen et al., 2014). This finding echoes the conclusions of numerous previous studies (Barrico et al., 2018; Kowarik et al., 2013; Riley et al., 2018).

Given the observed differences in woody plant diversity between artificial green spaces and remnant forests in urban mountain parks, it is advisable to design parks that conserve natural habitats while also accommodating their ornamental and recreational functions. Research underscores the crucial role of natural habitat preservation in species conservation (Fedrowitz et al., 2014; Zeller et al., 2023). Furthermore, natural habitats support the conservation of native or rare species, contribute to the restoration of species diversity, and promote structural heterogeneity on a localized scale (Chen et al., 2021). Incorporating native plants extensively into artificial green spaces is also recommended. This practice not only prevents the dominance of a few garden species, enriching species balance and diversity (Wang et al., 2021) but also ensures a reliable propagation source for native woody vegetation in urban settings (Kowarik et al., 2013). Additionally, using native plants enhances connectivity between artificial green spaces and remnant forests within parks, thereby facilitating species exchange and further supporting the conservation of native plant diversity (Chang et al., 2021).

Although our research contributes new insights into the impact of parkification and development duration on karst mountain forests, it is essential to recognize certain limitations. This study was confined to only three parks, which restricts the generalizability of our findings. Including a greater number and variety of parks with a broader range of development times would have provided a more comprehensive understanding of how park development influences woody plant diversity. Additionally, the diversity in management practices across the parks and the varying degrees of urbanization at the study sites could account for some of the observed differences in plant diversity. Despite these constraints, our findings offer valuable perspectives on the development of karst mountain parks and the conservation of urban plant diversity.

## 5 CONCLUSION

This study examined the diversity of woody plants in artificial green spaces and remnant forests within karst mountain parks, considering their development times. The research aimed to understand the impacts of time and land transformation into parkland on woody plant diversity in these distinct habitats. Our findings revealed significant disparities between the two environments. Artificial green spaces showed lower sapling/seedling regeneration, as well as higher homogeneity in plant composition compared with remnant forests. Notably, remnant forests were rich in native species, whereas artificial green spaces comprised mostly ornamental garden varieties. These insights highlight the ecological importance of incorporating native species during the transformation of remnant forests into urban mountain parks. Preserving native flora is essential for maintaining and enhancing urban plant biodiversity. By doing so, we can create more diverse and ecologically supportive environments that not only meet recreational needs but also play a crucial role in biodiversity conservation. Therefore, urban park development should include strategies to protect and integrate native species to foster a balanced and resilient urban ecosystem.

## AUTHOR CONTRIBUTIONS

**Weize Wang:** Investigation (equal); methodology (equal); software (equal); validation (equal); visualization (equal); writing – original draft (lead); writing – review and editing (equal). **Xiaoyan Gao:** Investigation (equal); methodology (equal); software (equal); writing – review and editing (equal). **Chunhua Cen:** Investigation (equal); methodology (equal); writing – review and editing (equal). **Mengping Jian:** Investigation (equal); methodology (equal); writing – review and editing (equal). **Zijin Wang:** Investigation (equal); methodology (equal); writing – review and editing (equal). **Jingyi Yang:** Conceptualization (lead); funding acquisition (lead); software (equal); supervision (lead); writing – review and editing (equal).

## CONFLICT OF INTEREST STATEMENT

The authors declare that they have no known competing financial interests or personal relationships that could have appeared to influence the work reported in this article.

## Data Availability

The data for this study are available via the Mendeley Data Repository. https://doi.org/10.17632/p3ztgnr7r4.1.
